# Specific protein kinase C isoform exerts chronic inhibition on the slowly activating delayed-rectifier potassium current by affecting channel trafficking

**DOI:** 10.1080/19336950.2021.1882112

**Published:** 2021-02-04

**Authors:** Xiangbo Gou, Tingting Hu, Yu Gou, Chaoqi Li, Ming Yi, Mengran Jia

**Affiliations:** a Tianjin Key Labortory of Drug Targeting and Bioimaging, Tianjin University of Technology, Tianjin, China; b School of Chemistry and Chemical Engineering, Tianjin University of Technology, Tianjin, China; c Department of Orthopaedic Surgery, Tianjin Hospital, Tianjin University, Tianjin, China; d Department of Neurobiology, School of Basic Medical Science, Advanced Innovation Center for Human Brain Protection, Capital Medical University, Beijing, China

**Keywords:** Protein kinase C, ion channel, *I*_ks_, KCNQ1, intracellular trafficking

## Abstract

The slowly activating delayed rectifier K^+^ current (*I*_Ks_) plays a key role in the repolarization of ventricular action potential in the human heart and is formed by the pore-forming α-subunit encoded by KCNQ1 (Kv7.1) and β-subunit encoded by KCNE1. Evidence suggested that *I*_Ks_ was regulated through protein kinase C (PKC) pathway, but the mechanism is controversial. This study was designed to identify the specific PKC isoform involved in the long-term regulation of *I*_Ks_ current. The *I*_Ks_ current was recorded using whole-cell patch-clamp technique in human embryonic kidney (HEK) 293B cell co-transfected with human KCNQ1/KCNE1 genes. The results revealed that both chronic activation of Ang II and PMA reduced the *I*_Ks_ current in a long-term regulation (about 24 hours). Further evidence showed that PKCε knockdown by siRNA antagonized the AngII-induced chronic inhibition on the *I*_Ks_ current, whereas knockdown of cPKC (PKCα and PKCβ) attenuated the inhibition effect of PMA on the current. Moreover, the forward transport inhibition of the channel with brefeldin A alleviated the Ang II-induced chronic inhibition on *I*_Ks_ current, while the channel endocytosis inhibition with dynasore alleviated both Ang II and PMA-induced chronic inhibition on *I*_Ks_ current. The above results showed that PKCε activation promoted the channel endocytosis and inhibited the channel forward transport to the plasma membrane, while cPKC activation only promoted the channel endocytosis, which both down regulated the channel current.

## Introduction

The delayed-rectifier potassium current including *I*_Ks_ and *I*_Kr_ is the major ion channel essential to the cardiac repolarization phase of the action potential in human and determines the action potential duration (APD) in cardiac [1]. *I*_Ks_ is formed by the pore-forming α-subunit encoded by KCNQ1 (Kv7.1) and β-subunit encoded by KCNE1. The reduction of *I*_Ks_ current caused by genetic mutations or drugs is linked to QT interval prolongation in the electrocardiogram (ECG), which can increase the risk of cardiac arrhythmia occurrence and sudden death; meanwhile, the congenital long-QT syndrome 1 (LQT1) patients are mainly related to the mutation of KCNQ1 gene [2]. As one of the repolarization reserves, *I*_Ks_ current becomes the main current to promote repolarization and prevent the arrhythmia occurrence when other repolarization reserves (such as *I*_Kr_) decrease or adrenergic levels increase in large animals [3]. Therefore, the prolonged APD is likely to cause torsade de pointes and increase the incidence of arrhythmia for the LQT1 patients with a down-regulated *I*_Ks_ function [4,5]. Therefore, it is of great importance to study the molecular mechanism of *I*_Ks_ downregulation in pathological conditions (i.e. cardiac hypertrophy and heart failure) for preventing the occurrence of arrhythmia. At present, the regulation of *I*_Ks_ current under pathological conditions has not been fully explained.

*I*_Ks_ is regulated by many factors such as nerves and body fluids, among which angiotensin II (AngII) is one of the main body fluids that cause hypertrophic remodeling and electrical remodeling of myocardial tissue [6,7]. Our previous studies showed that the QT interval was prolonged in isolated guinea pig hearts by AngII perfusion (200 nM), and further studies indicated that AngII exerted inhibition on the *I*_Ks_ current through protein kinase C (PKC) phosphorylating the channel in an acute regulation manner [8]. Phosphorylation is an effective way to regulate channel function and further affect cardiac electrophysiological characteristics; therefore, it also plays a vital role in the regulation of *I*_Ks_ current [9].

Chronic PKC activation has been implicated in many pathological conditions, such as cancer, lung and kidney diseases and especially heart failure [10–12]. It was reported that Gq-protein-coupled receptors (GqPCR) activation induced the KCNQ1 channel endocytosis by the conventional PKC (cPKC, including α, βI, βII, γ) [13], especially the PKC βII isoform involved in the reduction of KCNQ1 channel expression caused by GqPCR activation [14]. Our previous studies identified that AngⅡ exerted a stronger effect on novel PKC (PKCε) than cPKC (PKCα and PKCβ), while PMA exerted a stronger effect on cPKC inversely [8]. Under the pathological conditions, such as cardiac hypertrophy, PKC isoforms expression were significantly up-regulated, which promoted the development of cardiac hypertrophy [15]. Up to now, although we have investigated the acute effect of different PKC isoform on *I*_Ks_ current by different pathways, the chronic effect of specific PKC isoforms on *I*_Ks_ current is not clear. Thus, we will discuss the chronic effect of specific PKC isoforms (cPKC and PKCε) on *I*_Ks_ current. At present, the study was designed (1) to evaluate the chronic effect of specific PKC isoform on *I*_Ks_ current, (2) to define the distinct chronic effect of specific PKC isoform on *I*_Ks_ current using siRNA knockdown technology, and (3) to study the mechanism underlying the regulation *I*_Ks_ current by specific PKC isoform in a chronic regulation manner.

## Material and methods

### Solutions and chemicals

Ang II (Enzo Life Sciences) was prepared as a 1 mM stock solution in water. Bis-1 (Sigma) was prepared as a 5 mM stock solution in DMSO. PMA (Sigma) was prepared as a 2 mM stock solution in DMSO. PKCε peptide activator (εV1-2, EAVSLKPT), cPKC peptide activator (cPKC-AP, SVEIWD) and their scramble peptides were synthesized by LifeTein Biotechnology Company (Beijing, China), which were all prepared as 2 mM stock solutions in water. All peptide activators and scramble peptides were conjugated to a delivery peptide, oligo-arginine (R8), to make the peptide cell membrane permeable [16]. All stock solutions were stored at −20°C. The highest final concentration of DMSO in external solution was ≤ 0.1%, a concentration that had no effect on the current recording.

### Cell culture and transfection

Human embryonic kidney (HEK) 293B cells were cultured in 35 mm petri dishes in Dulbecco’s Modified Eagle’s Medium supplemented with 10% fetal bovine serum and 1% penicillin-streptomycin solution under 5% CO_2_ at 37°C. The cells were stably co-expressing KCNQ1/KCNE1 channel, while the human AT1 receptor cDNA (200 ng) was transiently transfected using a Lipofectamine 2000 Reagent kit (Invitrogen, USA) following the manufacturer’s instructions. Experiments were performed within 24 to 48 hours after the transfection.

### PKC isoenzyme knockdown

Double-stranded short interfering RNA (siRNA) targeting human PKCα (CGACTGGGAAAAACTGGAG), PKCβ (GAAGATGAACTCTTCCAAT) or PKCε (CTTCAAACCACGCATTAAA) (RIBOBIO, China) was transfected (100 nM) into HEK 293B cell stably co-expressing KCNQ1/KCNE1 genes using LipoRNAmax (Invitrogen, UK) according to the manufacturer’s protocol [8]. Transfection media was replaced with culture media 4 hours after transfection, and the experiments were performed 48 hours later.

### Electrophysiology

The *I*_Ks_ current was measured using a MultiClamp 700B amplifier (Molecular Devices, USA) at room temperature under continuous superfusion with the external solution. When filled with the pipette solution, the electrodes had a tip resistance between 0.5 and 2.5 MΩ. The electrical signals were sampled at 2.5–10 kHz and filtered at 1 kHz using a low-pass filter and digitized with an A/D converter (Digidata 1322, Molecular Devices, USA). The external solution contained (in mM): NaCl 140, MgCl_2_ 1, KCl 5.4, glucose 10, HEPES 10, and CaCl_2_ 2 (pH adjusted to 7.4 with NaOH). The pipette solution contained (in mM): KCl 140, HEPES 10, MgCl_2_ 1, and EGTA 5 (pH adjusted to 7.2 with KOH). A perforated patch configuration was used in HEK 293B cell to prevent *I*_Ks_ current rundown after the cell membrane ruptured, and the patch pipette was back-filled with amphotericin B (600 ng/ml). Following the patch perforation, the whole-cell membrane capacitance was measured from integration of the capacitive transients evoked by voltage steps from −50 mV to −60 mV, which did not activate any time-dependent membrane current. Series resistances were compensated 80% in order to minimize voltage errors. The pClamp software (Clampfit 10.4, Molecular Devices, USA) was used to generate voltage-pulse protocols and acquire data [8].

### Western blotting

The KCNQ1 membrane protein expression was evaluated by western blot analysis. The membrane protein was extracted with the Pierce Cell Surface Protein Isolation Kit (Thermo scientific). First, the cells were incubated with sulfo-NHs-SS-biotin marker dissolved in PBS for 30 minutes at 4°C. The above ubiquitination reaction was terminated by adding the Quenching Solution, and the supernatant was discarded by centrifugation. The adherent cells were scraped down and washed with TBS, and the lysate was added after the centrifugation. The supernatant was collected centrifugally and mixed with the washed NeutrAvidin Agarose and continuted to be incubated at room temperature for 1 hour in a shaking table. The DTT was diluted to a final concentration of 50 mM with sample buffer and incubated for 1 hour. The membrane protein was the filtrate collected by centrifugation and analyzed by western blot. The following primary antibodies were used as follows: anti-Na/K-ATPase (Proteintech, 1:500) and anti-KCNQ1 (Alomone, 1:700).

### Statistical analysis

The data were expressed as means±SEM. SPSS 19.0 software was used for the data analysis. Group comparisons were performed with paired or unpaired Student’s *t*-tests (for single two-group comparisons) and ANOVA with Dunnett’s post hoc tests (for multiple-group comparisons). Differences were considered significant if *P* < 0.05.

## Results

### *The chronic effect of Ang II on* I*_Ks_ current*

**Figure 1.**
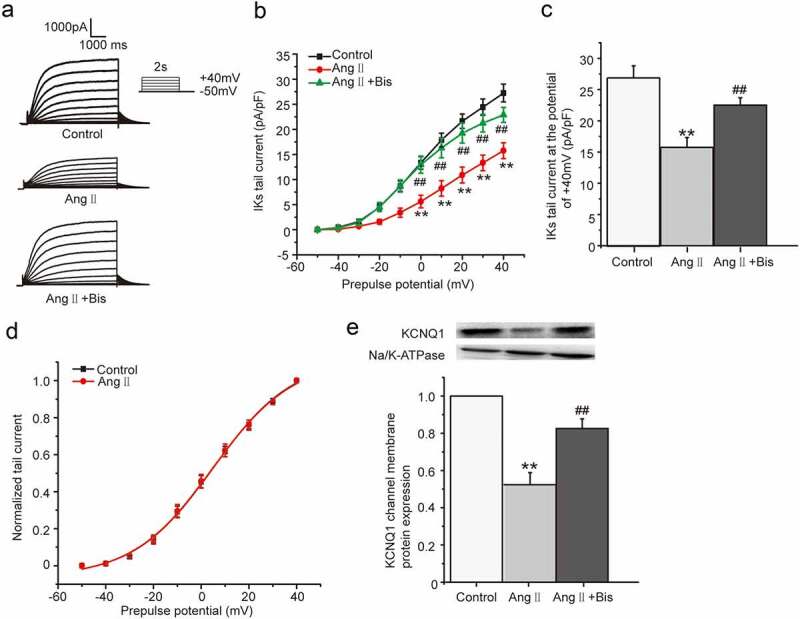
The chronic effect of Ang II on *I*_Ks_ current in HEK 293B cell. (a) The representative *I*_Ks_ current evoked by voltage protocol shown in inset under the Ang II (100 nM) treatment. (b) The current–voltage relationship for the tail currents under the Ang II (100 nM) treatment. (c) The summary data for the tail currents under the Ang II (100 nM) treatment at +40 mV prepulse. (d) The normalized I–V relationship for *I*_Ks_ current. The solid lines represent fits to a Boltzmann function. (e) The representative immunoblot and summary data for the KCNQ1 channel membrane protein under the Ang II (100 nM) treatment (**P < 0.01, compared with the control group; ^##^*P* < 0.01, compared with the Ang II treatment group)

As one of the main body fluids, AngII can cause hypertrophic remodeling and electrical remodeling of myocardial tissue. Our previous studies found that AngII produced the acute inhibition on *I*_Ks_ current through PKCε phosphorylating the channel directly [8]. But the electrical remodeling of myocardial tissue involved the chronic effect caused by PKC activation was not clearly understood. The HEK 293B cell stably co-expressing KCNQ1/KCNE1 genes transfected with the human AT1 receptor cDNA was used to investigate the chronic effect of Ang II on the *I*_Ks_ current. The cells were divided into three groups, including control group, Ang II group, and Ang II plus Bis-1 group, which were treated with normal medium, Ang II (100 nM) and Ang II (100 nM) plus Bis-1 (100 nM) for about 24 hours, respectively. The current was elicited from the holding potential of −80 mV to prepulses from −50 mV to +40 mV for a 5 s duration and was followed by a test pulse to −50 mV to evoke the slowly decaying outward tail currents. The results showed that compared with the control group, AngII markedly reduced both the depolarization and tail currents, which were alleviated in the presence of nonselective PKC inhibitor Bis-1 (Figure 1(a)). Moreover, the tail current density was significantly decreased at the potential range from 0 mV to +40 mV (*P* < 0.01, Figure 1(b)), with the tail current decreased about 42.19% by the AngII treatment at the potential of +40 mV (from 27.23 ± 1.77 pA/pF to 15.74 ± 1.57 pA/pF, *P* < 0.01, Figure 1(c)), which was alleviated by Bis-1 (*P* < 0.01, Figure 1(c)). The tail current amplitude normalized to the maximum tail current amplitude was used to construct the activation curve shown in Figure 1(d). When fit as a Boltzmann function, the half-maximum activation voltage (*V*_1/2_) and slope factor under control condition were 5.01 ± 1.32 mV and 16.75 ± 1.39, respectively, which were not significantly different from those after the AngII treatment (4.75 ± 1.47 mV for *V*_1/2_, 16.79 ± 1.57 for slope factor, *P* > 0.05). We next explored the effect of AngII on the KCNQ1 channel membrane protein expression which reflected the ion channel number. The membrane protein was extracted and used to detected the KCNQ1 channel membrane protein expression by western blot in all the groups. The results revealed that the KCNQ1 channel membrane protein expression decreased significantly after the AngII treatment (*P* < 0.01, Figure 1(e)), which suggested that AngII decreased the *I*_Ks_ current by reducing the KCNQ1 channel number not by changing the channel gating.

### *The chronic effect of PMA on* I*_Ks_ current*

**Figure 2.**
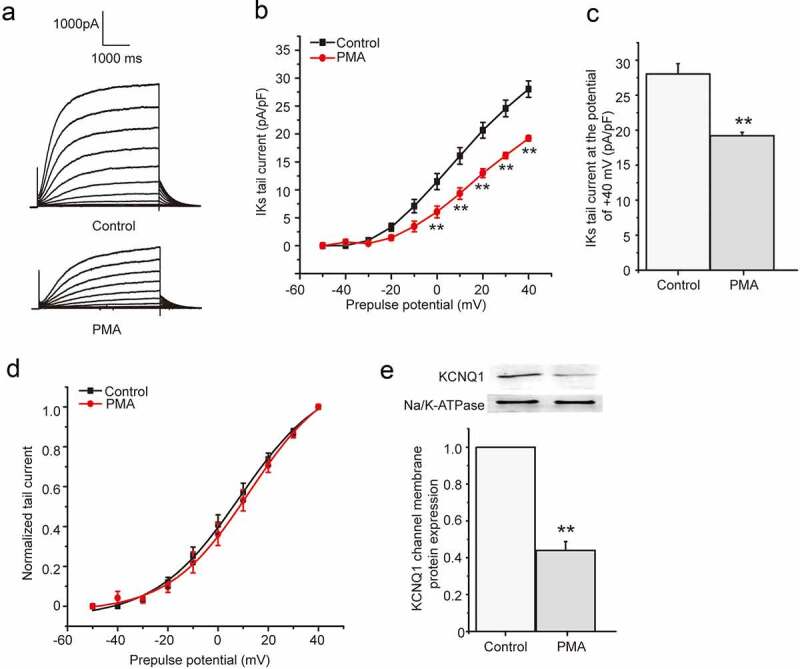
The chronic effect of PMA on *I*_Ks_ current in HEK 293B cell. (a) The representative *I*_Ks_ current under the PMA (100 nM) treatment. (b) The current–voltage relationship for the tail currents under the PMA (100 nM) treatment. (c) The summary data for the tail currents under the PMA (100 nM) treatment at +40 mV prepulse. (d) The normalized I–V relationship for *I*_Ks_ current. The solid lines represent fits to a Boltzmann function. (e) The representative immunoblot and summary data for the KCNQ1 channel membrane protein under the PMA (100 nM) treatment (**P < 0.01, compared with the control group)

We next tested the effect of phorbol ester PMA since it is widely used as a PKC activator. We investigated the chronic effect of PMA on *I*_Ks_ current in HEK 293B cell by activating PKC directly. The cells were divided into two groups, including control group and PMA group, which were treated with normal medium and PMA (100 nM) for about 24 hours, respectively. The results showed PMA markedly reduced both the depolarization and tail currents (Figure 2(a)), and the tail current density of *I*_Ks_ was significantly decreased at the potential range from 0 mV to +40 mV (*P* < 0.01, Figure 2(b)). Furthermore, the tail current decreased about 32% by the PMA treatment at the potential of +40 mV (from 28.03 ± 1.47 pA/pF to 19.21 ± 0.49 pA/pF, *P* < 0.01, Figure 2(c)). The tail current amplitude normalized to the maximum tail current amplitude was used to construct the activation curve shown in Figure 2(d). When fit as a Boltzmann function, the half-maximum activation voltage (*V*_1/2_) and slope factor under control conditions were 8.61 ± 1.51 mV and 16.84 ± 1.43, respectively, which were not significantly different from those after the PMA treatment (12.59 ± 1.85 mV for *V*_1/2_, 16.09 ± 1.50 for slope factor, *P* > 0.05). We next explored the effect of PMA on the KCNQ1 channel membrane protein expression. The membrane protein was extracted after the PMA treatment for 24 hours and then used to detect the KCNQ1 channel membrane protein expression by western blot. The results revealed that PMA treatment for 24 hours could significantly decrease the KCNQ1 channel membrane protein expression (about 56%, *P* < 0.01, Figure 2(e)), which suggested that PMA also decreased the *I*_Ks_ current by reducing the KCNQ1 channel number.

### *The chronic effect of different PKC isoform on* I*_Ks_ current*

**Figure 3.**
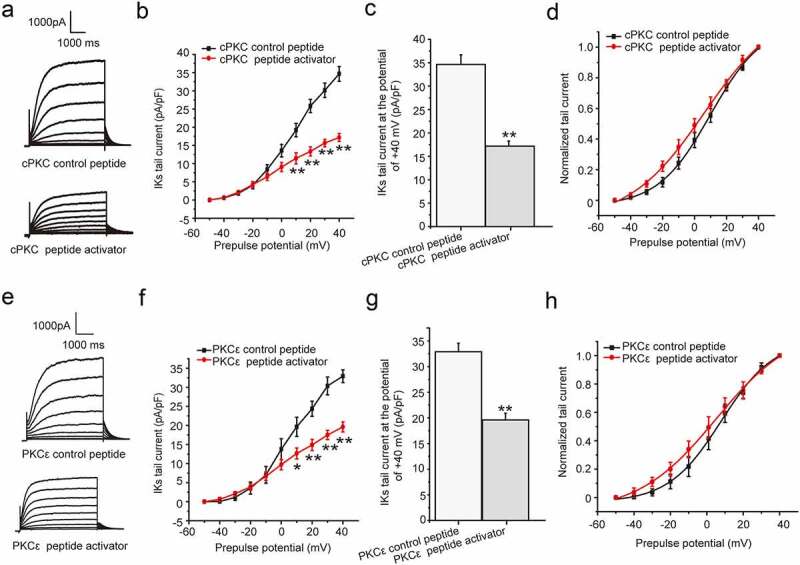
The chronic effect cPKC and PKCε activation on *I*_Ks_ current in HEK 293B cell. (a) The representative *I*_Ks_ current under the cPKC activation (200 nM). (b) The current–voltage relationship for the *I*_Ks_ tail currents under the cPKC activation (200 nM). (c) The summary data for the tail currents under the cPKC activation (200 nM) at +40 mV prepulse. (d) The normalized I–V relationship for *I*_Ks_ tail currents. The solid lines represent fits to a Boltzmann function. (e) The representative *I*_Ks_ current under the PKCε activation (200 nM). (f) The current–voltage relationship for the *I*_Ks_ tail currents under the PKCε activation (200 nM). (g) The summary data for the *I*_Ks_ tail currents under the PKCε activation (200 nM) at +40 mV prepulse. (h) The normalized I–V relationship for *I*_Ks_ tail currents. The solid lines represent fits to a Boltzmann function. (**P < 0.01, compared with the cPKC or PKCε control peptide group; *P < 0.05, compared with the cPKC or PKCε control peptide group)

We had confirmed that both PMA and Ang II exerted the chronic inhibition effect on *I*_Ks_ current by activation of PKC. We next investigated the effect of direct activation of specific PKC isoform (mainly cPKC and PKCε) on the current by different PKC peptide activators. The peptides added with R8-conjugated cell-permeable peptide selectively activate specific PKC isoform [17]. The different PKC isoform peptide activators and their control peptide were synthesized and used as previously reported [8]. The cells were divided into four groups, including cPKC control peptide group, cPKC peptide activator group, PKCε control peptide group, and PKCε peptide activator group, which were treated with cPKC control peptide (200 nM), cPKC peptide activator (200 nM), PKCε control peptide (200 nM), and PKCε peptide activator (200 nM) for about 24 hours, respectively. For the four groups above, the cPKC control peptide group and cPKC peptide activator group were used to investigate the effect of cPKC activation on the currrent, while the later two groups were used to investigate the effect of PKCε activation on the currrent. Compared with the control peptide, the results showed that both the depolarization and tail currents were significantly reduced after the cPKC and PKCε peptide activator treatment (Figure 3(a,e)), and the tail current density was all significantly inhibited at the potential range from +10 mV to +40 mV (*P* < 0.01 or *P* < 0.05, Figure 3(b,f)). Furthermore, the tail current decreased significantly at the potential range of +40 mV (cPKC control peptide: 34.66 ± 2.03 pA/pF, cPKC peptide activator: 17.18 ± 1.08 pA/pF, *P* < 0.01, Figure 3(c); PKCε control peptide: 32.91 ± 1.65 pA/pF, PKCε peptide activator: 19.60 ± 1.31 pA/pF, *P* < 0.01, Figure 3(g)). The tail current amplitude normalized to the maximum tail current amplitude was used to construct the activation curves shown in Figure 3(d and h). When fit as a Boltzmann function, the half-maximum activation voltage (*V*_1/2_) was 9.98 ± 1.08 mV for cPKC control peptide and 7.96 ± 1.10 mV for PKCε control peptide, while the slope factors were 16.47 ± 0.97 for cPKC control peptide and 14.54 ± 1.03 for PKCε control peptide, which were not significantly different from those in the presence of PKC peptide activators, the *V*_1/2_ value was 6.10 ± 2.23 mV for cPKC peptide activator and 5.11 ± 1.42 mV for PKCε peptide activator, while the slope factors were 23.65 ± 2.61 for cPKC peptide activator and 21.50 ± 1.67 for PKCε peptide activator (*P* > 0.05). The results confirmed that both chronic cPKC and PKCε activation elicited an inhibitory action on the *I*_Ks_ current.

### *The specific PKC isoform involved in the inhibition of Ang II and PMA on* I*_Ks_ current*

**Figure 4.**
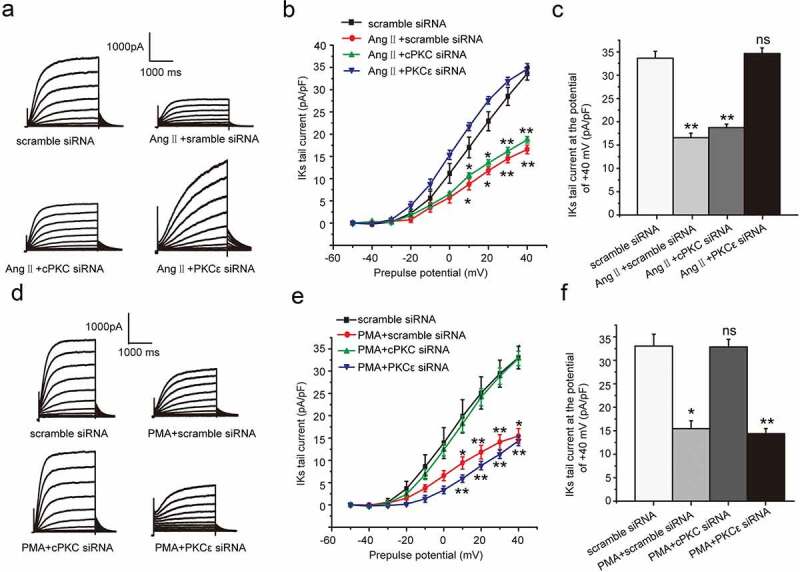
PKCε and cPKC involved in the chronic inhibition of Ang II and PMA on *I*_Ks_ current in HEK 293B cell. (a) The representative *I*_Ks_ current by Ang II treatment under the condition of different PKC isoform knocked-down. (b) The current–voltage relationship for the tail currents by Ang II treatment under the condition of different PKC isoform knocked-down. (c) The summary data for the tail currents by Ang II treatment under the condition of different PKC isoform knocked-down at +40 mV prepulse. (d) The representative *I*_Ks_ current by PMA treatment under the condition of different PKC isoform knocked-down. (e) The current–voltage relationship for the tail currents by PMA treatment under the condition of different PKC isoform knocked-down. (f) The summary data for the tail currents by PMA treatment under the condition of different PKC isoform knocked-down at +40 mV prepulse. (**P < 0.01, compared with the scramble siRNA group; *P < 0.05, compared with the scramble siRNA group)

In this part, we continued to discuss the specific PKC isoform involved in the chronic inhibition effect of AngII and PMA on the *I*_Ks_ current. We next explored which PKC isoform mediated the effect of Ang II or PMA by using a gene knockdown approach with custom-designed siRNAs targeting either cPKC (PKCα and PKCβ) or PKCε. To explore the inhibition effect of AngII on the *I*_Ks_ current, we treated the cells with scramble siRNA (100 nM), AngII (100 nM) plus scramble siRNA(100 nM), AngII plus cPKC siRNA, and AngII plus PKCε siRNA, respectively. As shown, the *I*_Ks_ current was significantly inhibited by the AngII treatment at the potential range from +10 mV to +40 mV, which was alleviated by the PKCε siRNA transfection, not by cPKC siRNA (scramble siRNA: 33.64 ± 1.48 mV, AngII+scramble siRNA: 16.57 ± 0.97 mV, AngII+cPKC siRNA: 18.74 ± 0.74 mV, AngII+PKCε siRNA: 34.64 ± 1.23 mV, *P* < 0.01 or *P < *0.05, Figure 4(a–c)). The results revealed that the chronic inhibition effect of AngII on the *I*_Ks_ current was regulated through PKCε.

We next discussed which PKC isoform involved in the chronic inhibition effect of PMA on *I*_Ks_ current. To explore the inhibition effect of PMA on the *I*_Ks_ current, we treated the cells with scramble siRNA (100 nM), PMA (100 nM) plus scramble siRNA (100 nM), PMA plus cPKC siRNA, and PMA plus PKCε siRNA, respectively. As shown, the *I*_Ks_ current was significantly inhibited under the PMA treatment at the potential range from +10 mV to +40 mV, which was alleviated by cPKC siRNA transfection, not by PKCε siRNA (scramble siRNA: 33.03 ± 2.53 mV, PMA+scramble siRNA: 15.45 ± 1.66 mV, PMA+cPKC siRNA: 32.86 ± 1.61 mV, PMA+PKCε siRNA: 14.38 ± 1.10 mV, *P* < 0.01 or *P* < 0.05, Figure 4(d–f)). The results revealed that the chronic inhibition effect of PMA on *I*_Ks_ current was regulated through cPKC.

### *The molecular mechanism underlying the chronic inhibition of Ang II and PMA on* I*_Ks_ current*

**Figure 5.**
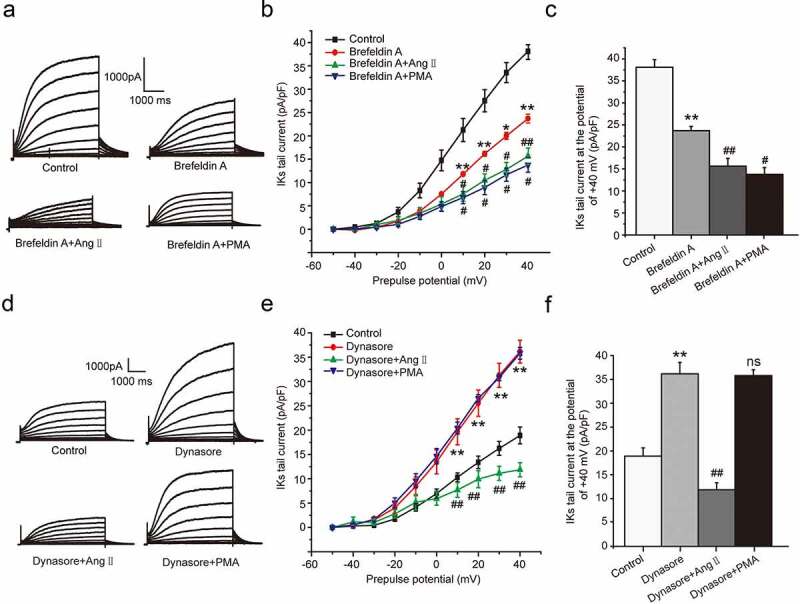
The molecular mechanism underlying the chronic inhibition of Ang II and PMA on *I*_Ks_ current in HEK 293B cell. (a) The representative *I*_Ks_ current by the Ang II and PMA treatment under the channel forward transport inhibition. (b) The current–voltage relationship for the *I*_Ks_ current by the Ang II and PMA treatment under the channel forward transport inhibition. (c) The summary data for the tail currents by the Ang II and PMA treatment under the channel forward transport inhibition at +40 mV prepulse. (d) The representative *I*_Ks_ current by the Ang II and PMA treatment under the channel endocytosis inhibition. (e) The current–voltage relationship for the *I*_Ks_ current by the Ang II and PMA treatment under the channel endocytosis inhibition. (f) The summary data for the *I*_Ks_ tail currents by the Ang II and PMA treatment under the channel endocytosis inhibition at +40 mV prepulse. (**P < 0.01, compared with the control group; ^##^*P* < 0.01, compared with Brefeldin A or Dynasore group; ^#^*P* < 0.05, compared with Brefeldin A group)

We further to investigate whether the ion channel transport was involved in the chronic inhibition of Ang II and PMA on *I*_Ks_ current. We divided the cells into four groups, including control group, brefeldin A group, brefeldin A plus AngII group, and brefeldin A plus PMA group. In the groups above, brefeldin A was used to inhibit the forward transport of ion channel by disrupting the trans-Golgi network [18]. As expected, the *I*_Ks_ current decreased significantly at the potential range from +10 mV to +40 mV (*P* < 0.01 or *P* < 0.05, Figure 5(a–c)) in the presence of brefeldin A. We next investigated the effect of PMA and AngII on *I*_Ks_ current in the presence of brefeldin A. The results indicated that both PMA and AngII further decreased the *I*_Ks_ current based on the effect of brefeldin A (*P* < 0.01 or *P* < 0.05, Figure 5(c)). The results illustrated that both PMA and AngII could accelerate the endocytosis of channel and lead to the current down-regulation.

We next investigated the effect of PMA and AngII on the channel forward transport. We divided the cells into four groups, including control group, dynasore group, dynasore plus AngII group, and dynasore plus PMA group. In the groups above, dynasore was used to inhibit the endocytosis of KCNQ1 channel as an endocytosis inhibitor [19]. The results showed the *I*_Ks_ current increased at the potential range from +10 mV to +40 mV (*P* < 0.01, Figure 5(d–f)) in the presence of dynasore, which was alleviated by the co-culture of AngII (*P* < 0.01, Figure 5(d–f)), not by PMA (*P* > 0.05, Figure 5(d–f)). The results elucidated that AngII could also inhibit the forward transport of the KCNQ1 channel and lead to the current reduction.

## Discussion

The aim of this study was to identify the specific PKC isoform participated in the chronic effect of AngII and PMA on the *I*_Ks_ current, as well as the mechanisms involved. In this research, we studied the chronic activation of cPKC and PKCε on the *I*_Ks_ current. Our data indicated that (1) both cPKC and PKCε chronic activation could inhibit the *I*_Ks_ current, (2) AngII exerted the inhibition on the *I*_Ks_ current by PKCε activation, while PMA exerted the inhibition by cPKC activation, (3) AngII inhibited the *I*_Ks_ current by facilitating the channel endocytosis as well as suppressing the channel forward transport process, and (4) PMA inhibited the *I*_Ks_ current only by facilitating the channel endocytosis.

There is increasing evidence that phosphorylation is an important way to regulate the channel function. There is no doubt that epinephrine β1 receptor stimulates and increases the *I*_Ks_ current through cAMP-PKA pathway [17]. Until now, we and others have shown the effect of acute PKC stimulation on the *I*_Ks_ current, but the long-term regulation of *I*_Ks_ current by PKC is undefined. GqPCRs such as adrenergic receptor (α1), angiotensin II receptor (AT1), and endothelin receptor (ET1) are known to mediate positive inotropism in human ventricular myocardium [18]. The Gq protein stimulates phosphatidylinositide-specific phospholipase C (PLC) after it is activated. The substrate of PLC is phosphatidylinositol 4, 5-biphosphate (PIP_2_) so that agonist stimulation of the receptor causes the reduction of PIP_2_ in the plasma membrane. Hydrolysis of PIP_2_ generates inositol 1, 4, 5-triphosphate (IP_3_) and diacylglycerol (DAG). Stimulation of IP_3_ receptor by IP_3_ releases Ca^2+^ from intracellular stores; furthermore, DAG activates PKC. Their stimulation contributes to the increase in contractility at the early stage of heart failure, but chronic stimulation of these receptors has been shown to be associated with myocardial hypertrophy and remodeling. We and others also reported that KCNQ1/KCNE1 channel phosphorylation was contributed to the acute GqPCR-mediated activation of the channel *via* PKC [8,19]. In contrast to the inhibition effect on the *I*_Ks_ current of AT1 receptor activation, our results showed that direct PKC activation by PMA increased the *I*_Ks_ current acutely [8]. More and more evidences show that activation of different GqPCRs lead to the activation of different PKC isoform [20]. It was reported that only PKCα, βI, βII, and PKCε isoforms translocated to the cell membrane when the adrenergic α1 receptor excited, while all the PKC isoforms translocated to the cell membrane under the PMA action [21]. The above reports suggest that different PKC agonists lead to activation of different PKC isoforms (whether PKC is activated directly or indirectly), which play biological functions by translocating to the cell membrane. Moreover, due to the different activation and transposition, different PKC isoforms display different regulation on the channel.

It was reported that PKC isoforms expression including PKCα, PKCβ, and PKCε were upregulated significantly under the pathological conditions such as cardiac hypertrophy [22,23]. Chronic stimulation of PKC has been shown to be associated with myocardial hypertrophy and remodeling [15]. Our recent data showed that Ang II and PMA decreased the *I*_Ks_ current, which was achieved by decreasing the channel number not the channel dynamics. By using specific siRNA knockdown method, we provided further evidence that cPKC (PKCα and PKCβ) underlined the down-regulation of *I*_Ks_ current by PMA, while PKCε selectively attenuated the inhibitory action of Ang II on the *I*_Ks_ current.

The ion channel number is depended on the intracellular trafficking, which includes the forward and reverse transport process. The forward transport is the progress that after the gene transcription, the channel proteins fold and mature on the endoplasmic reticulum and Golgi, which are finally wrapped by secretory vesicles and transported to the cell membrane to perform functions [24]. Reverse transport refers to the endocytosis and degradation progress after the channel proteins invaginated through the cell membrane [25]. For the experimental results above, we speculated that activation of different PKC isoforms may affect the forward transport or reverse transport of the channel, ultimately affecting the channel function. Therefore, we used the endocytosis inhibitor dynasore and forward transport inhibitor brefeldin A to inhibit the reverse and forward transport process, consequently determined the way by which the cPKC and PKCε isoforms inhibited the *I*_Ks_ current. Our results revealed that PKCε activation exerted a chronic inhibiton on the channel by promoting the channel endocytosis and inhibiting the channel forward transport, while cPKC exerted chronic inhibiton on the channel by promoting channel endocytosis only. Both the forward transport and endocytic degradation processes of the channel involve Rab proteins, which are small GTPases. Rab proteins exist in the cytoplasm as an inactive form binded to GDP (Rab-GDP), but exist as an active form binded to GTP (Rab-GTP) on the cell membrane and transport vesicles. Rab is one of the important factors regulating the channel transport and function [26]. After entering into the early endosome through Rab5, the channel proteins have four ways to go: (1) transport to cell membrane through Rab4 (rapid recycling), (2) degradation by proteasome, (3) degradation by lysosomal through Rab7, and (4) circulation to cell membrane through Rab11 (slow recycling) [27]. Rab4, Rab5, Rab7, and Rab11 are widely expressed in the heart [13], which are all involved in the KCNQ1/KCNE1 channel transport in the cell [25,28]. We hypothesize whether different PKC isoforms affect the channel transport through different Rab proteins, which we will research in our future work.

## Conclusion

Collectively, we show that Ang II-stimulated PKCε activation promoted the endocytosis of KCNQ1/KCNE1 channel and inhibited the channel forward transport to the plasma membrane, while PMA-stimulated cPKC activation only promoted the internalization of KCNQ1/KCNE1 channel. To our knowledge, this is the first time to show that different PKC isoform inhibit the *I*_Ks_ current by affecting different progress of the ion channel intracellular trafficking. This study is of great significance to reveal the molecular mechanism of cardiac hypertrophy electrical remodeling and to find new targets for arrhythmia.

## Limitations

Our data had identified that specific PKC isoform suppressed *I*_Ks_ in a long-term manner by affecting different channel trafficking process, which may involve direct modulation of the channel, or potentially other signaling proteins.

## References

[1] Moss AJ, Kass RS. Long QT syndrome: from channels to cardiac arrhythmias. J Clin Invest. 2005 8;115(8):2018–2024. 16075042 10.1172/JCI25537 PMC1180552

[2] Splawski I, Shen J, Timothy KW, et al. Spectrum of mutations in long-QT syndrome genes. KVLQT1, HERG, SCN5A, KCNE1, and KCNE2. Circulation. 2000 9 5;102(10):1178–1185. 10973849 10.1161/01.cir.102.10.1178

[3] Hegyi B, Bossuyt J, Ginsburg KS, et al. Altered repolarization reserve in failing rabbit ventricular myocytes: calcium and beta-adrenergic effects on delayed- and inward-rectifier potassium currents. Circ Arrhythm Electrophysiol. 2018 2;11(2):e005852. 29437761 10.1161/CIRCEP.117.005852 PMC5813707

[4] Schwartz PJ, Priori SG, Spazzolini C, et al. Genotype-phenotype correlation in the long-QT syndrome: gene-specific triggers for life-threatening arrhythmias. Circulation. 2001 1 2;103(1):89–95. 11136691 10.1161/01.cir.103.1.89

[5] Wu J, Naiki N, Ding WG, et al. A molecular mechanism for adrenergic-induced long QT syndrome. J Am Coll Cardiol. 2014 3 4;63(8):819–827. 24184248 10.1016/j.jacc.2013.08.1648

[6] Fischer R, Dechend R, Gapelyuk A, et al. Angiotensin II-induced sudden arrhythmic death and electrical remodeling. Am J Physiol Heart Circ Physiol. 2007 8;293(2):H1242–53. 17416596 10.1152/ajpheart.01400.2006

[7] Yasuno S, Kuwahara K, Kinoshita H, et al. Angiotensin II type 1a receptor signalling directly contributes to the increased arrhythmogenicity in cardiac hypertrophy. Br J Pharmacol. 2013 12;170(7):1384–1395. 23937445 10.1111/bph.12328 PMC3838685

[8] Gou X, Wang W, Zou S, et al. Protein kinase C epsilon mediates the inhibition of angiotensin II on the slowly activating delayed-rectifier potassium current through channel phosphorylation. J Mol Cell Cardiol. 2018 3;116:165–174. 29452158 10.1016/j.yjmcc.2018.02.010

[9] Dai S, Hall DD, Hell JW. Supramolecular assemblies and localized regulation of voltage-gated ion channels. Physiol Rev. 2009 4;89(2):411–452. 19342611 10.1152/physrev.00029.2007 PMC2733249

[10] Mochly-Rosen D, Das K, Grimes KV. Protein kinase C, an elusive therapeutic target? Nat Rev Drug Discov. 2012 12;11(12):937–957. 23197040 10.1038/nrd3871 PMC3760692

[11] Li J, Gobe G. Protein kinase C activation and its role in kidney disease. Nephrology. 2006 10;11(5):428–434. 17014557 10.1111/j.1440-1797.2006.00673.x

[12] Arimoto T, Takeishi Y, Takahashi H, et al. Cardiac-specific overexpression of diacylglycerol kinase zeta prevents Gq protein-coupled receptor agonist-induced cardiac hypertrophy in transgenic mice. Circulation. 2006 1 3;113(1):60–66. 16380548 10.1161/CIRCULATIONAHA.105.560771

[13] Parks XX, Ronzier E, OU J, et al. Fluvastatin inhibits Rab5-mediated IKs internalization caused by chronic Ca(2+)-dependent PKC activation. J Mol Cell Cardiol. 2019 4;129:314–325. 30898664 10.1016/j.yjmcc.2019.03.016 PMC6645917

[14] Liu X, Wang Y, Zhang H, et al. Different protein kinase C isoenzymes mediate inhibition of cardiac rapidly activating delayed rectifier K(+) current by different G-protein coupled receptors. Br J Pharmacol. 2017 12;174(23):4464–4477. 28941256 10.1111/bph.14049 PMC5715974

[15] Singh RM, Cummings E, Pantos C, et al. Protein kinase C and cardiac dysfunction: a review. Heart Fail Rev. 2017 11;22(6):843–859. 28702857 10.1007/s10741-017-9634-3 PMC5635086

[16] Chen L, Wright LR, Chen CH, et al. Molecular transporters for peptides: delivery of a cardioprotective epsilonPKC agonist peptide into cells and intact ischemic heart using a transport system, R(7). Chem Biol. 2001 12;8(12):1123–1129. 11755391 10.1016/s1074-5521(01)00076-x

[17] Harmati G, Banyasz T, Barandi L, et al. Effects of beta-adrenoceptor stimulation on delayed rectifier K(+) currents in canine ventricular cardiomyocytes. Br J Pharmacol. 2011 2;162(4):890–896. 20973780 10.1111/j.1476-5381.2010.01092.x PMC3042199

[18] Port JD, Bristow MR. Altered beta-adrenergic receptor gene regulation and signaling in chronic heart failure. J Mol Cell Cardiol. 2001 5;33(5):887–905. 11343413 10.1006/jmcc.2001.1358

[19] Matavel A, Lopes CM. PKC activation and PIP(2) depletion underlie biphasic regulation of IKs by Gq-coupled receptors. J Mol Cell Cardiol. 2009 5;46(5):704–712. 19233191 10.1016/j.yjmcc.2009.02.006 PMC2668609

[20] Shirai Y, Saito N. Activation mechanisms of protein kinase C: maturation, catalytic activation, and targeting. J Biochem. 2002 11;132(5):663–668. 12417013 10.1093/oxfordjournals.jbchem.a003271

[21] OU J, Sorenson J, Jhun BS, et al. Isoform-specific dynamic translocation of PKC by alpha1-adrenoceptor stimulation in live cells. Biochem Biophys Res Commun. 2015 9 25;465(3):464–470. 26277396 10.1016/j.bbrc.2015.08.040 PMC4564329

[22] Konopatskaya O, Poole AW. Protein kinase Calpha: disease regulator and therapeutic target. Trends Pharmacol Sci. 2010 1;31(1):8–14. 19969380 10.1016/j.tips.2009.10.006 PMC2809215

[23] Ferreira JC, Brum PC, Mochly-Rosen D. betaIIPKC and epsilonPKC isozymes as potential pharmacological targets in cardiac hypertrophy and heart failure. J Mol Cell Cardiol. 2011 10;51(4):479–484. 21035454 10.1016/j.yjmcc.2010.10.020 PMC3135714

[24] de Git KC, de Boer TP, Vos MA, et al. Cardiac ion channel trafficking defects and drugs. Pharmacol Ther. 2013 7;139(1):24–31. 23558293 10.1016/j.pharmthera.2013.03.008

[25] Seebohm G, Strutz-Seebohm N, Birkin R, et al. Regulation of endocytic recycling of KCNQ1/KCNE1 potassium channels. Circ Res. 2007 3 16;100(5):686–692. 17293474 10.1161/01.RES.0000260250.83824.8f

[26] Hutagalung AH, Novick PJ. Role of Rab GTPases in membrane traffic and cell physiology. Physiol Rev. 2011 1;91(1):119–149. 21248164 10.1152/physrev.00059.2009 PMC3710122

[27] Wang T, Cheng Y, Dou Y, et al. Trafficking of an endogenous potassium channel in adult ventricular myocytes. Am J Physiol Cell Physiol. 2012 11 1;303(9):C963–76. 22914645 10.1152/ajpcell.00217.2012 PMC3492822

[28] Piccini I, Fehrmann E, Frank S, et al. Adrenergic stress protection of human iPS cell-derived cardiomyocytes by Fast Kv7.1 recycling. Front physiol. 2017;8:705. 28959214 10.3389/fphys.2017.00705 PMC5603700

